# Mid-arm muscle circumference as a substantial factor against mortality among people with elevated gamma gaps

**DOI:** 10.18632/oncotarget.19372

**Published:** 2017-07-19

**Authors:** Yuan-Ping Chao, Yi-Fen Lai, Tung-Wei Kao, Tao-Chun Peng, Yuan-Yung Lin, Mu-Tsun Shih, Wei-Liang Chen, Li-Wei Wu

**Affiliations:** ^1^ Division of Family Medicine, Department of Family and Community Medicine, Tri-Service General Hospital and School of Medicine, National Defense Medical Center, Taipei, Taiwan, Republic of China; ^2^ Division of Geriatric Medicine, Department of Family and Community Medicine, Tri-Service General Hospital and School of Medicine, National Defense Medical Center, Taipei, Taiwan, Republic of China; ^3^ Department of Otolaryngology–Head and Neck Surgery, Tri-Service General Hospital and School of Medicine, National Defense Medical Center, Taipei, Taiwan, Republic of China; ^4^ Graduate Institute of Medical Sciences, National Defense Medical Center, Taipei, Taiwan, Republic of China; ^5^ Division of Urology, Department of Surgery, Tri-Service General Hospital and School of Medicine, National Defense Medical Center, Taipei, Taiwan, Republic of China

**Keywords:** anthropometric parameters, MAMC, gamma gap, NHANES, mortality

## Abstract

Gamma gap is the difference in total serum proteins and albumin and an elevated gamma gap is related to infections, malignancy, and rheumatic diseases. An elevated gamma gap is also associated with higher mortality due to the correlation with inflammatory status. The study aimed to utilize mid-arm muscle circumference (MAMC) to assist in predicting all-cause mortality, cancer mortality, and cardiovascular mortality in people with elevated gamma gaps. Data were obtained from the third U.S. National Health and Nutrition Examination Survey (1988–1994), which contained 14,011 adults aged 20 to 90 years during up to 14.3 years of follow-up. The Primary analysis examined MAMC in tertiles and revealed the demographic and characteristics of the study population. Receiver operating characteristic curve analysis was used and the most suitable cut-off point of gamma gap was 3.65 g/dl. The secondary analysis employed Cox proportional hazards models stratified by age, gender and body mass index to evaluate the hazard ratios for all-cause mortality, cancer mortality, and cardiovascular mortality associated with the MAMC. As the MAMC tertiles increased in group with gamma gap ≥ 3.65 g/dl, individuals with elder age (60–90 years), normal range of body mass index (19–24.9 kg/m^2^), and male gender tended to have lower hazard ratios for all-cause mortality, cancer mortality, and cardiovascular mortality. These substantial findings indicate that higher MAMC may be a protective factor of all cause-mortality, cancer mortality, and cardiovascular mortality among older male with normal body mass index and elevated gamma gaps.

## INTRODUCTION

The definition of gamma gap is the difference in total serum proteins and albumin, and a value above 3.5 or 4.0 g/dl is considered to be elevated. An elevated gamma gap was indicative of infections, malignancy, or other generalized inflammation. Previous literature has shown strong correlation between gamma gap and mortality, which may be due to gamma gap characterizing inflammatory status [1]. Among those with high levels of gamma gap, having adequate cardiorespiratory fitness is of importance in reducing mortality [2]. Of additional interest to the present study is a less investigated factor related to mortality among people with an elevated gamma gap, the mid-arm muscle circumference (MAMC). The MAMC is calculated as: mid-upper arm circumference (MAC) (cm) − 0.3142 x triceps skinfold (TS) thickness (mm), which is a single most portable and simple measurement. It is salient to establish the contributions of the widely applicable, convenient, and inexpensive anthropometric indices as well as potential mechanisms linking anthropometric indices to mortality to inform the development of specific prevention and intervention strategies in people with elevated gamma gaps. The aim of this investigation is to explore the potential protective effects of the MAMC on mortality in people with elevated gamma gaps. The National Health and Nutrition Examination Survey (NHANES) III database was adopted in this study. We assessed the associations of MAMC and all-cause mortality, cancer mortality, and cardiovascular (CV) mortality among people with and without elevated gamma gaps, respectively.

## RESULTS

### Preliminary analysis

The study included 14,011 adults with 6,688 (47.7%) male and 7,323 (52.3%) female participants. The mean MAMC was 25.9 ± 3.9 cm and the mean age was 47.8 ± 19.1 years. In our analytical cohort study, 3,432 deaths occurred during a mean follow-up of 14.3 years, including 1,898 male and 1,534 female.

### Study sample characteristics

Table 1 presented the demographic and clinical characteristics of the study population by MAMC tertiles. Of the participants, higher levels of body mass index (BMI), systolic blood pressure (SBP), diastolic blood pressure (DBP), serum total cholesterol (TC), serum total triglycerides (TG), serum glucose, serum low-density lipoprotein (LDL), serum uric acid (UA), aspartate aminotransferase (AST), alanine aminotransferase (ALT), serum total bilirubin, and serum albumin were observed in higher MAMC tertiles. However, higher gamma gap, serum high-density lipoprotein (HDL), and C-reactive protein (CRP) levels were shown in the lower MAMC tertiles. Male participants increased as female participants decreased in the higher MAMC tertiles. Asthma and smoking tended to increase in the higher MAMC tertiles while other cancer had an opposite trend. Higher percentages of non-Hispanic white, type 2 diabetes mellitus (DM), stroke, and congestive heart failure (CHF) were presented in the higher MAMC tertiles. Nonetheless, the maximal percentage of skin cancer was in the second tertile of MAMC.

**Table 1:** Characteristics of the study participants by mid-arm muscle circumference tertiles

Characteristics of the study participants	Tertiles of mid-arm muscle circumference (cm)			Total *n* = 14,011	*P* for trend
	T1 (18.0-27.2 cm)	T2 (27.3-29.5 cm)	T3 (29.6-40.0 cm)		
	*n* = 4,670	*n* = 4,670	*n* = 4,671		
**Continuous variables**^**a**^					
MAMC (cm), mean (SE)	21.673 (1.410)	25.739 (1.161)	30.373 (2.081)	25.929 (3.897)	< 0.001
Gamma Gap (g/dL), mean (SE)	3.259 (0.477)	3.258 (0.481)	3.223 (0.482)	3.247 (0.480)	< 0.001
Age (years), mean (SE)	46.130 (19.994)	50.840 (19.972)	46.360 (16.829)	47.780 (19.112)	< 0.001
BMI (kg/m^2^), mean (SE)	24.010 (4.012)	27.130 (4.889)	29.290 (5.254)	26.810 (5.218)	< 0.001
SBP (mmHg), mean (SE)	120.170 (23.101)	127.830 (22.694)	128.020 (18.885)	125.340 (21.947)	< 0.001
DBP (mmHg), mean (SE)	68.110 (13.085)	72.280 (12.836)	76.780 (12.030)	72.390 (13.144)	< 0.001
Serum TG (mg/dL), mean (SE)	122.090 (96.320)	144.880 (106.942)	166.730 (137.438)	144.580 (116.335)	< 0.001
Serum total cholesterol (mg/dL), mean (SE)	203.190 (45.959)	207.310 (45.771)	207.320 (42.813)	205.940 (44.909)	< 0.001
Serum HDL-cholesterol (mg/dL), mean (SE)	56.860 (15.856)	50.770 (15.387)	45.960 (13.731)	51.200 (15.667)	< 0.001
Serum LDL-cholesterol (mg/dL), mean (SE)	122.130 (38.758)	129.190 (38.832)	132.060 (37.862)	127.810 (38.707)	< 0.001
Serum glucose (mg/dL), mean (SE)	94.500 (33.250)	102.180 (38.038)	102.820 (38.649)	100.17 (36.947)	< 0.001
Serum CRP (mg/dL), mean (SE)	0.452 (0.836)	0.500 (0.781)	0.437 (0.663)	0.463 (0.764)	< 0.001
Serum UA (mg/dL), mean (SE)	4.509 (1.258)	5.422 (1.389)	6.122 (1.372)	5.451 (1.495)	< 0.001
AST (U/L), mean (SE)	20.080 (12.203)	22.520 (18.028)	24.730 (16.610)	22.440 (15.922)	< 0.001
ALT (U/L), mean (SE)	14.280 (13.059)	17.320 (15.723)	22.490 (20.143)	18.030 (16.910)	< 0.001
Serum total bilirubin (umol/L), mean (SE)	0.529 (0.301)	0.590 (0.319)	0.667 (0.365)	0.595 (0.334)	< 0.001
Serum albumin (g/dL), mean (SE)	4.081 (0.372)	4.135 (0.383)	4.216 (0.358)	4.144 (0.375)	< 0.001
**Categorical variables**^**b**^					
Gender (male), *N* (%)	272 (5.8)	2314 (49.6)	4102 (87.8)	6688 (47.7)	< 0.001
Non-Hispanic white, *N* (%)	1195 (25.6)	1320 (28.3)	1300 (27.8)	3815 (27.2)	< 0.001
Type 2 diabetes mellitus, *N* (%)	250 (5.4)	429 (9.2)	409 (8.8)	1088 (7.8)	< 0.001
Malignancy, *N* (%)	203 (4.3)	201 (4.3)	117 (2.5)	521 (3.7)	< 0.001
Stroke, *N* (%)	97 (2.1)	156 (3.3)	100 (2.1)	353 (2.5)	< 0.001
Congestive heart failure, *N* (%)	107 (2.3)	207 (4.4)	167 (3.6)	481 (3.4)	< 0.001
Asthma, *N* (%)	300 (6.4)	323 (6.9)	345 (7.4)	968 (6.9)	0.251
Smoking, *N* (%)	76 (1.6)	520 (11.1)	983 (21.0)	1579 (11.3)	< 0.001

Abbreviations:

N, number; SE, standard errors; MAMC, mid-arm muscle circumference; BMI, body mass index; SBP, systolic blood pressure; DBP, diastolic blood pressure; Serum TG, serum total triglycerides; HDL, high-density lipoprotein; LDL, low-density lipoprotein; Serum CRP, serum C-reactive protein; Serum UA, serum uric acid; AST, aspartate aminotransferase; ALT, alanine aminotransferase.

^a^Values were expressed as mean (standard errors).

^b^Values in the categorical variables were expressed as number (%).

### The cut-off point of the gamma gap

To further investigated the relationship between the MAMC and all-cause mortality, cancer mortality, and CV mortality, we divided the participants into 2 groups based on the gamma gap level. The P value of the interaction test between MAMC and gamma gap was < 0.001. The receiver operating characteristic (ROC) curves of gamma gap for detecting all-cause mortality, cancer mortality, and CV mortality was performed. ROC curve was used for predicting the ethnic-specific cut-off points of gamma gap and the area under curve (AUC) was calculated with 95% confidence interval (CI). The optimal AUC (95% CI) was 0.534 (0.523 – 0.545) for all-cause mortality, 0.533 (0.512 – 0.555) for cancer mortality, and 0.528 (0.512 – 0.544) for CV mortality when gamma gap was 3.65 g/dl. The three mortality outcomes had identical cut-off value of gamma gap; therefore, we divided the participants into groups with gamma gap < 3.65 g/dl and ≥ 3.65 g/dl.

### Association between the MAMC and all-cause mortality, cancer mortality, and cardiovascular mortality

Cox proportional hazards models stratified by age, BMI, and gender were utilized to determine the hazard ratios (HRs) for all-cause mortality, cancer mortality, and CV mortality associated with the MAMC in the 2 gamma gap groups. Multivariable adjusted analyses were conducted and the results were shown in Tables 2–10.

**Table 2:** Cox proportional hazards regression of all-cause mortality for mid-arm muscle circumference stratified by age in the US individuals

GammaGap < 3.65 (g/dL)				GammaGap ≥ 3.65 (g/dL)			
Models ^a^	Tertiles of MAMC	Hazard Ratio (95% CI)	*P* -value	Models ^a^	Tertiles of MAMC	Hazard Ratio (95% CI)	*P* -value
**Aged 20–39 years**				**Aged 20–39 years**			
Model 1	T2 v.s. T1 T3 v.s. T1	1.77 (1.12–2.81) 2.66 (1.75–4.04)	0.015 < 0.001	Model 1	T2 v.s. T1 T3 v.s. T1	1.69 (0.88–3.23) 1.81 (0.94–3.48)	0.112 0.076
Model 2	T2 v.s. T1 T3 v.s. T1	1.32 (0.75–2.33) 1.58 (0.83–3.00)	0.330 0.159	Model 2	T2 v.s. T1 T3 v.s. T1	1.31 (0.65–2.65) 0.94 (0.38–2.33)	0.457 0.891
Model 3	T2 v.s. T1 T3 v.s. T1	1.34 (0.76–2.37) 1.66 (0.87–3.19)	0.309 0.126	Model 3	T2 v.s. T1 T3 v.s. T1	1.48 (0.71–3.09) 0.98 (0.39–2.47)	0.299 0.960
Model 4	T2 v.s. T1 T3 v.s. T1	1.34 (0.76–2.37) 1.65 (0.86–3.17)	0.312 0.132	Model 4	T2 v.s. T1 T3 v.s. T1	1.43 (0.68–3.01) 0.97 (0.38–2.48)	0.341 0.950
**Aged 40–59 years**				**Aged 40–59 years**			
Model 1	T2 v.s. T1 T3 v.s. T1	1.64 (1.24–2.16) 1.36 (1.04–1.78)	<0.001 0.026	Model 1	T2 v.s. T1 T3 v.s. T1	1.59 (1.02–2.49) 1.44 (0.91–2.26)	0.040 0.117
Model 2	T2 v.s. T1 T3 v.s. T1	1.20 (0.88–1.65) 0.88 (0.60–1.27)	0.253 0.481	Model 2	T2 v.s. T1 T3 v.s. T1	1.15 (0.71–1.85) 0.69 (0.40–1.20)	0.567 0.186
Model 3	T2 v.s. T1 T3 v.s. T1	1.21 (0.88–1.66) 0.88 (0.61–1.28)	0.233 0.513	Model 3	T2 v.s. T1 T3 v.s. T1	1.18 (0.72–1.92) 0.77 (0.43–1.36)	0.510 0.370
Model 4	T2 v.s. T1 T3 v.s. T1	1.21 (0.88–1.66) 0.88 (0.60–1.28)	0.240 0.492	Model 4	T2 v.s. T1 T3 v.s. T1	1.14 (0.70–1.87) 0.77 (0.43–1.38)	0.596 0.382
**Aged 60–90 years**				**Aged 60–90 years**			
Model 1	T2 v.s. T1 T3 v.s. T1	1.17 (1.06–1.30) 0.88 (0.78–0.99)	0.002 0.034	Model 1	T2 v.s. T1 T3 v.s. T1	0.89 (0.73–1.07) 0.89 (0.72–1.09)	0.220 0.268
Model 2	T2 v.s. T1 T3 v.s. T1	1.02 (0.91–1.15) 0.91 (0.79–1.06)	0.744 0.223	Model 2	T2 v.s. T1 T3 v.s. T1	0.74 (0.60–0.90) 0.71 (0.56–0.91)	0.004 0.008
Model 3	T2 v.s. T1 T3 v.s. T1	1.01 (0.9–1.14) 0.9 (0.78–1.05)	0.839 0.186	Model 3	T2 v.s. T1 T3 v.s. T1	0.74 (0.60–0.91) 0.67 (0.52–0.86)	0.005 0.002
Model 4	T2 v.s. T1 T3 v.s. T1	1.01 (0.9–1.14) 0.90 (0.78–1.05)	0.864 0.170	Model 4	T2 v.s. T1 T3 v.s. T1	0.75 (0.61–0.93) 0.69 (0.54–0.89)	0.007 0.004

^a^Adjusted covariates:

Model 1 = Unadjusted.

Model 2 = Model 1 + gender, race and body mass index (BMI).

Model 3 = Model 2 + serum high-density lipoprotein (HDL), serum fasting glucose, serum total cholesterol, serum total bilirubin, serum aspartate aminotransferase (AST).

Model 4 = Model 3 + history of congestive heart failure (CHF), stroke, malignancy and smoking.

**Table 3:** Cox proportional hazards regression of all-cause mortality for mid-arm muscle circumference stratified by body mass index (BMI) in the US individuals

GammaGap < 3.65 (g/dL)				GammaGap ≥ 3.65 (g/dL)			
Models ^a^	Tertiles of MAMC	Hazard Ratio (95% CI)	*P* -value	Models ^a^	Tertiles of MAMC	Hazard Ratio (95% CI)	*P* -value
**BMI 19–24.9 (kg/m**^**2**^**)**				**BMI 19–24.9 (kg/m**^**2**^**)**			
Model 1	T2 v.s. T1 T3 v.s. T1	1.51 (1.33–1.73) 0.64 (0.52–0.79)	< 0.001 < 0.001	Model 1	T2 v.s. T1 T3 v.s. T1	1.58 (1.23–2.01) 0.71 (0.48–1.06)	< 0.001 0.094
Model 2	T2 v.s. T1 T3 v.s. T1	0.87 (0.73–1.04) 0.80 (0.61–1.05)	0.134 0.108	Model 2	T2 v.s. T1 T3 v.s. T1	0.70 (0.51–0.96) 0.52 (0.32–0.84)	0.026 0.007
Model 3	T2 v.s. T1 T3 v.s. T1	0.89 (0.74–1.07) 0.83 (0.63–1.08)	0.202 0.173	Model 3	T2 v.s. T1 T3 v.s. T1	0.67 (0.49–0.92) 0.51 (0.31–0.81)	0.014 0.005
Model 4	T2 v.s. T1 T3 v.s. T1	0.90 (0.75–1.08) 0.84 (0.64–1.09)	0.250 0.189	Model 4	T2 v.s. T1 T3 v.s. T1	0.66 (0.47–0.91) 0.52 (0.32–0.84)	0.012 0.008
**BMI 25–29.9 (kg/m**^**2**^**)**				**BMI 25–29.9 (kg/m**^**2**^**)**			
Model 1	T2 v.s. T1 T3 v.s. T1	1.97 (1.67–2.33) 0.95 (0.80–1.13)	< 0.001 0.564	Model 1	T2 v.s. T1 T3 v.s. T1	2.08 (1.52–2.83) 1.82 (1.31–2.52)	< 0.001 < 0.001
Model 2	T2 v.s. T1 T3 v.s. T1	1.09 (0.90–1.31) 0.83 (0.66–1.06)	0.382 0.138	Model 2	T2 v.s. T1 T3 v.s. T1	1.06 (0.75–1.50) 0.98 (0.64–1.52)	0.725 0.943
Model 3	T2 v.s. T1 T3 v.s. T1	1.08 (0.90–1.31) 0.83 (0.65–1.05)	0.401 0.127	Model 3	T2 v.s. T1 T3 v.s. T1	1.04 (0.74–1.48) 0.89 (0.57–1.37)	0.811 0.595
Model 4	T2 v.s. T1 T3 v.s. T1	1.07 (0.89–1.30) 0.82 (0.64–1.04)	0.464 0.105	Model 4	T2 v.s. T1 T3 v.s. T1	1.06 (0.74–1.50) 0.86 (0.55–1.33)	0.753 0.495
**BMI ≥ 30 (kg/m**^**2**^**)**				**BMI ≥ 30 (kg/m**^**2**^**)**			
Model 1	T2 v.s. T1 T3 v.s. T1	1.39 (1.00–1.93) 1.63 (1.19–2.24)	0.049 0.002	Model 1	T2 v.s. T1 T3 v.s. T1	1.57 (0.94–2.61) 2.19 (1.34–3.59)	0.082 0.002
Model 2	T2 v.s. T1 T3 v.s. T1	1.23 (0.89–1.72) 1.34 (0.94–1.91)	0.214 0.110	Model 2	T2 v.s. T1 T3 v.s. T1	0.99 (0.60–1.66) 1.17 (0.69–2.00)	0.983 0.558
Model 3	T2 v.s. T1 T3 v.s. T1	1.24 (0.89–1.73) 1.34 (0.94–1.92)	0.197 0.104	Model 3	T2 v.s. T1 T3 v.s. T1	0.97 (0.58–1.63) 1.11 (0.65–1.90)	0.921 0.693
Model 4	T2 v.s. T1 T3 v.s. T1	1.25 (0.90–1.74) 1.37 (0.96–1.96)	0.185 0.084	Model 4	T2 v.s. T1 T3 v.s. T1	0.99 (0.59–1.66) 1.20 (0.70–2.05)	0.973 0.503

^a^Adjusted covariates:

Model 1 = Unadjusted.

Model 2 = Model 1 + age, gender, and race.

Model 3 = Model 2 + serum high-density lipoprotein (HDL), serum fasting glucose, serum total cholesterol, serum total bilirubin, serum aspartate aminotransferase (AST).

Model 4 = Model 3 + history of congestive heart failure (CHF), stroke, malignancy and smoking.

**Table 4:** Cox proportional hazards regression of all-cause mortality for mid-arm muscle circumference stratified by gender in the US individuals

GammaGap < 3.65 (g/dL)				GammaGap ≥ 3.65 (g/dL)			
Models ^a^	Tertiles of MAMC	Hazard Ratio (95% CI)	*P* -value	Models ^a^	Tertiles of MAMC	Hazard Ratio (95% CI)	*P* -value
Male				Male			
Model 1	T2 v.s. T1 T3 v.s. T1	0.49 (0.40–0.60) 0.24 (0.20–0.30)	< 0.001 < 0.001	Model 1	T2 v.s. T1 T3 v.s. T1	0.47 (0.34–0.64) 0.22 (0.16–0.30)	< 0.001 < 0.001
Model 2	T2 v.s. T1 T3 v.s. T1	0.76 (0.62–0.94) 0.59 (0.48–0.73)	0.010 < 0.001	Model 2	T2 v.s. T1 T3 v.s. T1	0.67 (0.49–0.92) 0.49 (0.35–0.68)	0.012 < 0.001
Model 3	T2 v.s. T1 T3 v.s. T1	0.78 (0.63–0.95) 0.61 (0.49–0.75)	0.014 < 0.001	Model 3	T2 v.s. T1 T3 v.s. T1	0.65 (0.47–0.89) 0.46 (0.33–0.64)	0.007 < 0.001
Model 4	T2 v.s. T1 T3 v.s. T1	0.78 (0.63–0.95) 0.61 (0.49–0.75)	0.015 < 0.001	Model 4	T2 v.s. T1 T3 v.s. T1	0.60 (0.44–0.83) 0.44 (0.31–0.61)	0.002 < 0.001
Female				Female			
Model 1	T2 v.s. T1 T3 v.s. T1	1.39 (1.22–1.57) 1.62 (1.31–2.00)	< 0.001 < 0.001	Model 1	T2 v.s. T1 T3 v.s. T1	1.06 (0.85–1.33) 1.58 (1.18–2.11)	0.602 0.002
Model 2	T2 v.s. T1 T3 v.s. T1	1.06 (0.94–1.21) 1.46 (1.18–1.81)	0.334 0.001	Model 2	T2 v.s. T1 T3 v.s. T1	0.83 (0.66–1.03) 1.23 (0.92–1.65)	0.096 0.156
Model 3	T2 v.s. T1 T3 v.s. T1	1.05 (0.93–1.19) 1.43 (1.15–1.77)	0.435 0.001	Model 3	T2 v.s. T1 T3 v.s. T1	0.80 (0.64–1.01) 1.21 (0.90–1.62)	0.062 0.208
Model 4	T2 v.s. T1 T3 v.s. T1	1.05 (0.92–1.19) 1.41 (1.14–1.75)	0.049 0.002	Model 4	T2 v.s. T1 T3 v.s. T1	0.82 (0.65–1.03) 1.26 (0.93–1.69)	0.091 0.131

^a^ Adjusted covariates:

Model 1 = Unadjusted.

Model 2 = Model 1 + age, race and body mass index (BMI).

Model 3 = Model 2 + serum high-density lipoprotein (HDL), serum fasting glucose, serum total cholesterol , serum total bilirubin, serum aspartate aminotransferase (AST).

Model 4 = Model 3 + history of congestive heart failure (CHF), stroke, malignancy and smoking.

**Table 5:** Cox proportional hazards regression of cancer mortality for mid-arm muscle circumference stratified by age in the US individuals

GammaGap < 3.65 (g/dL)				GammaGap ≥ 3.65 (g/dL)			
Models ^a^	Tertiles of MAMC	Hazard Ratio (95% CI)	*P* -value	Models ^a^	Tertiles of MAMC	Hazard Ratio (95% CI)	*P* -value
**Aged 20–39 years**				**Aged 20–39 years**			
Model 1	T2 v.s. T1 T3 v.s. T1	0.86 (0.39–1.92) 0.60 (0.25–1.41)	0.721 0.241	Model 1	T2 v.s. T1 T3 v.s. T1	1.45 (0.36–5.81) 1.31 (0.29–5.86)	0.598 0.726
Model 2	T2 v.s. T1 T3 v.s. T1	1.16 (0.49–2.74) 1.11 (0.31–3.95)	0.740 0.869	Model 2	T2 v.s. T1 T3 v.s. T1	1.27 (0.30–5.40) 1.06 (0.14–7.88)	0.746 0.954
Model 3	T2 v.s. T1 T3 v.s. T1	1.28 (0.53–3.10) 1.39 (0.38–5.12)	0.587 0.622	Model 3	T2 v.s. T1 T3 v.s. T1	1.27 (0.29–5.48) 1.09 (0.15–8.09)	0.752 0.935
Model 4	T2 v.s. T1 T3 v.s. T1	1.28 (0.53–3.10) 1.39 (0.38–5.14)	0.586 0.621	Model 4	T2 v.s. T1 T3 v.s. T1	1.37 (0.32–5.94) 1.14 (0.16–8.16)	0.673 0.897
**Aged 40–59 years**				**Aged 40–59 years**			
Model 1	T2 v.s. T1 T3 v.s. T1	1.52 (1.02–2.28) 1.04 (0.69–1.55)	0.040 0.863	Model 1	T2 v.s. T1 T3 v.s. T1	1.08 (0.47–2.45) 0.90 (0.38–2.12)	0.863 0.812
Model 2	T2 v.s. T1 T3 v.s. T1	1.14 (0.72–1.80) 0.72 (0.41–1.25)	0.569 0.240	Model 2	T2 v.s. T1 T3 v.s. T1	0.86 (0.35–2.11) 0.53 (0.18–1.58)	0.742 0.255
Model 3	T2 v.s. T1 T3 v.s. T1	1.16 (0.74–1.84) 0.75 (0.43–1.32)	0.519 0.320	Model 3	T2 v.s. T1 T3 v.s. T1	0.98 (0.39–2.47) 0.67 (0.22–2.00)	0.960 0.468
Model 4	T2 v.s. T1 T3 v.s. T1	1.17 (0.74–1.85) 0.76 (0.44–1.34)	0.495 0.346	Model 4	T2 v.s. T1 T3 v.s. T1	1.08 (0.42–2.79) 0.67 (0.22–2.05)	0.873 0.482
**Aged 60–90 years**				**Aged 60–90 years**			
Model 1	T2 v.s. T1 T3 v.s. T1	1.54 (1.20–1.96) 1.34 (1.03–1.73)	0.001 0.028	Model 1	T2 v.s. T1 T3 v.s. T1	1.09 (0.73–1.64) 1.17 (0.76–1.78)	0.678 0.472
Model 2	T2 v.s. T1 T3 v.s. T1	1.11 (0.85–1.46) 0.90 (0.65–1.25)	0.448 0.521	Model 2	T2 v.s. T1 T3 v.s. T1	0.60 (0.38–0.95) 0.49 (0.29–0.83)	0.028 0.008
Model 3	T2 v.s. T1 T3 v.s. T1	1.11 (0.84–1.46) 0.90 (0.64–1.25)	0.457 0.517	Model 3	T2 v.s. T1 T3 v.s. T1	0.66 (0.42–1.04) 0.53 (0.31–0.88)	0.073 0.015
Model 4	T2 v.s. T1 T3 v.s. T1	1.11 (0.84–1.47) 0.87 (0.62–1.21)	0.446 0.404	Model 4	T2 v.s. T1 T3 v.s. T1	0.66 (0.42–1.04) 0.57 (0.33–0.96)	0.076 0.036

^a^ Adjusted covariates:

Model 1 = Unadjusted.

Model 2 = Model 1 + gender, race and body mass index (BMI).

Model 3 = Model 2 + serum high-density lipoprotein (HDL), serum fasting glucose, serum total cholesterol , serum total bilirubin, serum aspartate aminotransferase (AST).

Model 4 = Model 3 + history of congestive heart failure (CHF), stroke, malignancy and smoking.

**Table 6:** Cox proportional hazards regression of cancer mortality for mid-arm muscle circumference stratified by body mass index (BMI) in the US individuals

GammaGap < 3.65 (g/dL)				GammaGap ≥ 3.65 (g/dL)			
Models ^a^	Tertiles of MAMC	Hazard Ratio (95% CI)	*P* -value	Models ^a^	Tertiles of MAMC	Hazard Ratio (95% CI)	*P* -value
**BMI 19–24.9 (kg/m**^**2**^**)**				**BMI 19–24.9 (kg/m**^**2**^**)**			
Model 1	T2 v.s. T1 T3 v.s. T1	1.67 (1.27–2.20) 0.91 (0.61–1.34)	< 0.001 0.630	Model 1	T2 v.s. T1 T3 v.s. T1	2.06 (1.24–3.42) 1.09 (0.53–2.21)	0.005 0.816
Model 2	T2 v.s. T1 T3 v.s. T1	0.95 (0.63–1.43) 0.94 (0.55–1.61)	0.815 0.825	Model 2	T2 v.s. T1 T3 v.s. T1	0.30 (0.15–0.63) 0.27 (0.11–0.69)	0.001 0.006
Model 3	T2 v.s. T1 T3 v.s. T1	0.99 (0.66–1.48) 1.01 (0.59–1.72)	0.961 0.985	Model 3	T2 v.s. T1 T3 v.s. T1	0.29 (0.13–0.61) 0.26 (0.10–0.66)	0.001 0.005
Model 4	T2 v.s. T1 T3 v.s. T1	1.00 (0.66–1.51) 0.99 (0.58–1.70)	0.995 0.973	Model 4	T2 v.s. T1 T3 v.s. T1	0.29 (0.13–0.63) 0.24 (0.09–0.63)	0.002 0.004
**BMI 25–29.9 (kg/m**^**2**^**)**				**BMI 25–29.9 (kg/m**^**2**^**)**			
Model 1	T2 v.s. T1 T3 v.s. T1	2.45 (1.72–3.51) 1.23 (0.85–1.77)	< 0.001 0.278	Model 1	T2 v.s. T1 T3 v.s. T1	2.14 (1.17–3.92) 2.21 (1.20–4.07)	0.013 0.011
Model 2	T2 v.s. T1 T3 v.s. T1	1.20 (0.80–1.79) 0.69 (0.42–1.13)	0.372 0.138	Model 2	T2 v.s. T1 T3 v.s. T1	0.74 (0.36–1.51) 0.66 (0.28–1.57)	0.406 0.352
Model 3	T2 v.s. T1 T3 v.s. T1	1.24 (0.83–1.84) 0.73 (0.45–1.19)	0.289 0.210	Model 3	T2 v.s. T1 T3 v.s. T1	0.77 (0.39–1.53) 0.64 (0.28–1.48)	0.458 0.300
Model 4	T2 v.s. T1 T3 v.s. T1	1.22 (0.82–1.82) 0.69 (0.42–1.13)	0.330 0.140	Model 4	T2 v.s. T1 T3 v.s. T1	0.77 (0.39–1.55) 0.66 (0.28–1.52)	0.467 0.327
**BMI ≥ 30 (kg/m**^**2**^**)**				**BMI ≥ 30 (kg/m**^**2**^**)**			
Model 1	T2 v.s. T1 T3 v.s. T1	1.65 (0.80–3.38) 2.10 (1.05–4.18)	0.172 0.035	Model 1	T2 v.s. T1 T3 v.s. T1	2.46 (0.73–8.24) 3.04 (0.92–10.04)	0.145 0.068
Model 2	T2 v.s. T1 T3 v.s. T1	1.52 (0.74–3.12) 1.59 (0.73–3.48)	0.258 0.246	Model 2	T2 v.s. T1 T3 v.s. T1	1.32 (0.39–4.48) 1.38 (0.39–4.96)	0.654 0.618
Model 3	T2 v.s. T1 T3 v.s. T1	1.50 (0.73–3.09) 1.48 (0.68–3.23)	0.272 0.324	Model 3	T2 v.s. T1 T3 v.s. T1	1.36 (0.40–4.65) 1.33 (0.37–4.78)	0.624 0.659
Model 4	T2 v.s. T1 T3 v.s. T1	1.51 (0.73–3.12) 1.46 (0.67–3.19)	0.261 0.345	Model 4	T2 v.s. T1 T3 v.s. T1	1.21 (0.35–4.21) 1.33 (0.37–4.77)	0.766 0.666

^a^ Adjusted covariates:

Model 1 = Unadjusted.

Model 2 = Model 1 + age, gender, and race.

Model 3 = Model 2 + serum high-density lipoprotein (HDL), serum fasting glucose, serum total cholesterol , serum total bilirubin, serum aspartate aminotransferase (AST).

Model 4 = Model 3 + history of congestive heart failure (CHF), stroke, malignancy and smoking.

**Table 7:** Cox proportional hazards regression of cancer mortality for mid-arm muscle circumference stratified by gender in the US individuals

GammaGap < 3.65 (g/dL)				GammaGap ≥ 3.65 (g/dL)			
Models^a^	Tertiles of MAMC	Hazard Ratio (95% CI)	*P* -value	Models ^a^	Tertiles of MAMC	Hazard Ratio (95% CI)	*P* -value
**Male**				**Male**			
Model 1	T2 v.s. T1 T3 v.s. T1	0.59 (0.37–0.96) 0.33 (0.21–0.53)	0.032 < 0.001	Model 1	T2 v.s. T1 T3 v.s. T1	0.39 (0.21–0.73) 0.15 (0.08–0.29)	0.003 < 0.001
Model 2	T2 v.s. T1 T3 v.s. T1	0.94 (0.58–1.51) 0.69 (0.43–1.12)	0.793 0.130	Model 2	T2 v.s. T1 T3 v.s. T1	0.37 (0.20–0.70) 0.26 (0.13–0.50)	0.002 < 0.001
Model 3	T2 v.s. T1 T3 v.s. T1	1.03 (0.64–1.67) 0.78 (0.48–1.26)	0.903 0.309	Model 3	T2 v.s. T1 T3 v.s. T1	0.46 (0.23–0.92) 0.32 (0.15–0.65)	0.027 0.002
Model 4	T2 v.s. T1 T3 v.s. T1	1.01 (0.62–1.63) 0.74 (0.46–1.21)	0.975 0.233	Model 4	T2 v.s. T1 T3 v.s. T1	0.44 (0.22–0.88) 0.32 (0.15–0.67)	0.021 0.002
**Female**				**Female**			
Model 1	T2 v.s. T1 T3 v.s. T1	1.57 (1.22–2.04) 1.41 (0.86–2.31)	0.001 0.169	Model 1	T2 v.s. T1 T3 v.s. T1	1.02 (0.62–1.66) 1.76 (0.96–3.25)	0.952 0.069
Model 2	T2 v.s. T1 T3 v.s. T1	1.14 (0.88–1.48) 1.14 (0.70–1.87)	0.320 0.597	Model 2	T2 v.s. T1 T3 v.s. T1	0.74 (0.45–1.22) 1.32 (0.72–2.43)	0.236 0.376
Model 3	T2 v.s. T1 T3 v.s. T1	1.13 (0.87–1.46) 1.08 (0.66–1.78)	0.375 0.757	Model 3	T2 v.s. T1 T3 v.s. T1	0.74 (0.45–1.22) 1.37 (0.74–2.54)	0.239 0.320
Model 4	T2 v.s. T1 T3 v.s. T1	1.13 (0.87–1.47) 1.08 (0.65–1.77)	0.345 0.773	Model 4	T2 v.s. T1 T3 v.s. T1	0.73 (0.44–1.22) 1.42 (0.76–2.64)	0.231 0.273

^a^ Adjusted covariates:

Model 1 = Unadjusted.

Model 2 = Model 1 + age, race and body mass index (BMI).

Model 3 = Model 2 + serum high-density lipoprotein (HDL), serum fasting glucose, serum total cholesterol , serum total bilirubin, serum aspartate aminotransferase (AST).

Model 4 = Model 3 + history of congestive heart failure (CHF), stroke, malignancy and smoking.

**Table 8:** Cox proportional hazards regression of cardiovascular mortality for mid-arm muscle circumference stratified by age in the US individuals

GammaGap < 3.65 (g/dL)				GammaGap ≥ 3.65 (g/dL)			
Models ^a^	Tertiles of MAMC	Hazard Ratio (95% CI)	*P* -value	Models ^a^	Tertiles of MAMC	Hazard Ratio (95% CI)	*P* -value
**Aged 20–39 years**				**Aged 20–39 years**			
Model 1	T2 v.s. T1 T3 v.s. T1	5.75 (1.24–26.60) 10.56 (2.46–45.33)	0.025 0.002	Model 1	T2 v.s. T1 T3 v.s. T1	0.97 (0.16–5.83) 3.22 (0.81–12.89)	0.977 0.098
Model 2	T2 v.s. T1 T3 v.s. T1	7.22 (1.48–35.31) 16.46 (3–90.26.00)	0.015 0.001	Model 2	T2 v.s. T1 T3 v.s. T1	0.37 (0.04–3.49) 0.52 (0.04–6.08)	0.388 0.600
Model 3	T2 v.s. T1 T3 v.s. T1	6.92 (1.4–34.17) 14.39 (2.55–81.08)	0.018 0.003	Model 3	T2 v.s. T1 T3 v.s. T1	0.44 (0.04–4.31) 0.54 (0.04–6.66)	0.481 0.634
Model 4	T2 v.s. T1 T3 v.s. T1	6.98 (1.41–34.42) 14.08 (2.49–79.55)	0.017 0.003	Model 4	T2 v.s. T1 T3 v.s. T1	0.39 (0.04–3.87) 0.39 (0.03–5.83)	0.420 0.495
**Aged 40–59 years**				**Aged 40–59 years**			
Model 1	T2 v.s. T1 T3 v.s. T1	2.19 (1.28–3.72) 2.28 (1.38–3.78)	0.004 0.001	Model 1	T2 v.s. T1 T3 v.s. T1	1.73 (0.78–3.83) 2.00 (0.93–4.33)	0.175 0.077
Model 2	T2 v.s. T1 T3 v.s. T1	1.46 (0.80–2.67) 1.27 (0.64–2.50)	0.214 0.494	Model 2	T2 v.s. T1 T3 v.s. T1	1.39 (0.60–3.21) 1.13 (0.44–2.91)	0.445 0.805
Model 3	T2 v.s. T1 T3 v.s. T1	1.47 (0.81–2.69) 1.28 (0.64–2.53)	0.209 0.484	Model 3	T2 v.s. T1 T3 v.s. T1	1.32 (0.56–3.07) 1.13 (0.43–2.97)	0.525 0.807
Model 4	T2 v.s. T1 T3 v.s. T1	1.41 (0.77–2.58) 1.19 (0.60–2.37)	0.268 0.613	Model 4	T2 v.s. T1 T3 v.s. T1	1.13 (0.48–2.69) 1.05 (0.39–2.84)	0.774 0.927
**Aged 60–90 years**				**Aged 60–90 years**			
Model 1	T2 v.s. T1 T3 v.s. T1	1.20 (1.03–1.38) 0.85 (0.72–1.00)	0.016 0.054	Model 1	T2 v.s. T1 T3 v.s. T1	0.81 (0.61–1.07) 0.76 (0.56–1.04)	0.136 0.089
Model 2	T2 v.s. T1 T3 v.s. T1	1.07 (0.91–1.25) 0.99 (0.80–1.22)	0.443 0.913	Model 2	T2 v.s. T1 T3 v.s. T1	0.68 (0.50–0.92) 0.65 (0.44–0.94)	0.012 0.022
Model 3	T2 v.s. T1 T3 v.s. T1	1.05 (0.89–1.24) 0.97 (0.78–1.20)	0.547 0.770	Model 3	T2 v.s. T1 T3 v.s. T1	0.68 (0.50–0.92) 0.62 (0.42–0.91)	0.013 0.013
Model 4	T2 v.s. T1 T3 v.s. T1	1.06 (0.90–1.25) 0.97 (0.78–1.20)	0.486 0.750	Model 4	T2 v.s. T1 T3 v.s. T1	0.67 (0.49–0.91) 0.62 (0.42–0.91)	0.011 0.014

^a^ Adjusted covariates:

Model 1 = Unadjusted.

Model 2 = Model 1 + gender, race and body mass index (BMI).

Model 3 = Model 2 + serum high-density lipoprotein (HDL), serum fasting glucose, serum total cholesterol , serum total bilirubin, serum aspartate aminotransferase (AST).

Model 4 = Model 3 + history of congestive heart failure (CHF), stroke, malignancy and smoking.

**Table 9:** Cox proportional hazards regression of cardiovascular mortality for mid-arm muscle circumference stratified by body mass index (BMI) in the US individuals

GammaGap < 3.65 (g/dL)				GammaGap ≥ 3.65 (g/dL)			
Models ^a^	Tertiles of MAMC	Hazard Ratio (95% CI)	*P* -value	Models ^a^	Tertiles of MAMC	Hazard Ratio (95% CI)	*P* -value
**BMI 19–24.9 (kg/m**^**2**^**)**				**BMI 19–24.9 (kg/m**^**2**^**)**			
Model 1	T2 v.s. T1 T3 v.s. T1	1.62 (1.33–1.97) 0.48 (0.34–0.70)	< 0.001 < 0.001	Model 1	T2 v.s. T1 T3 v.s. T1	1.46 (0.99–2.16) 0.39 (0.18–0.86)	0.056 0.019
Model 2	T2 v.s. T1 T3 v.s. T1	1.08 (0.84–1.39) 0.98 (0.64–1.50)	0.539 0.913	Model 2	T2 v.s. T1 T3 v.s. T1	0.53 (0.32–0.90) 0.45 (0.18–1.15)	0.018 0.094
Model 3	T2 v.s. T1 T3 v.s. T1	1.09 (0.85–1.41) 1.00 (0.65–1.54)	0.481 0.993	Model 3	T2 v.s. T1 T3 v.s. T1	0.49 (0.28–0.84) 0.42 (0.16–1.07)	0.010 0.069
Model 4	T2 v.s. T1 T3 v.s. T1	1.12 (0.87–1.44) 1.01 (0.65–1.56)	0.373 0.964	Model 4	T2 v.s. T1 T3 v.s. T1	0.45 (0.25–0.80) 0.40 (0.15–1.05)	0.006 0.061
**BMI 25–29.9 (kg/m**^**2**^**)**				**BMI 25–29.9 (kg/m**^**2**^**)**			
Model 1	T2 v.s. T1 T3 v.s. T1	2.02 (1.58–2.57) 0.88 (0.68–1.15)	< 0.001 0.348	Model 1	T2 v.s. T1 T3 v.s. T1	1.92 (1.20–3.08) 1.45 (0.87–2.43)	0.007 0.153
Model 2	T2 v.s. T1 T3 v.s. T1	0.94 (0.71–1.24) 0.78 (0.55–1.11)	0.674 0.172	Model 2	T2 v.s. T1 T3 v.s. T1	0.98 (0.58–1.64) 0.65 (0.32–1.30)	0.930 0.224
Model 3	T2 v.s. T1 T3 v.s. T1	0.94 (0.71–1.25) 0.78 (0.54–1.11)	0.680 0.165	Model 3	T2 v.s. T1 T3 v.s. T1	0.96 (0.57–1.61) 0.63 (0.31–1.27)	0.864 0.200
Model 4	T2 v.s. T1 T3 v.s. T1	0.92 (0.69–1.21) 0.75 (0.52–1.07)	0.543 0.117	Model 4	T2 v.s. T1 T3 v.s. T1	0.93 (0.55–1.59) 0.60 (0.29–1.22)	0.793 0.156
**BMI ≥ 30 (kg/m**^**2**^**)**				**BMI ≥ 30 (kg/m**^**2**^**)**			
Model 1	T2 v.s. T1 T3 v.s. T1	1.42 (0.89–2.26) 1.63 (1.04–2.54)	0.142 0.033	Model 1	T2 v.s. T1 T3 v.s. T1	1.47 (0.71–3.03) 2.20 (1.09–4.43)	0.302 0.028
Model 2	T2 v.s. T1 T3 v.s. T1	1.22 (0.76–1.95) 1.33 (0.79–2.22)	0.404 0.279	Model 2	T2 v.s. T1 T3 v.s. T1	0.81 (0.39–1.68) 1.07 (0.50–2.27)	0.571 0.862
Model 3	T2 v.s. T1 T3 v.s. T1	1.23 (0.77–1.98) 1.35 (0.81–2.25)	0.379 0.251	Model 3	T2 v.s. T1 T3 v.s. T1	0.89 (0.43–1.86) 1.10 (0.52–2.34)	0.761 0.800
Model 4	T2 v.s. T1 T3 v.s. T1	1.22 (0.76–1.95) 1.38 (0.83–2.31)	0.416 0.213	Model 4	T2 v.s. T1 T3 v.s. T1	0.95 (0.45–2.00) 1.34 (0.62–2.88)	0.895 0.454

^a^ Adjusted covariates:

Model 1 = Unadjusted.

Model 2 = Model 1 + age, gender, and race.

Model 3 = Model 2 + serum high-density lipoprotein (HDL), serum fasting glucose, serum total cholesterol , serum total bilirubin, serum aspartate aminotransferase (AST).

Model 4 = Model 3 + history of congestive heart failure (CHF), stroke, malignancy and smoking.

**Table 10:** Cox proportional hazards regression of cardiovascular mortality for mid-arm muscle circumference stratified by gender in the US individuals

GammaGap < 3.65 (g/dL)				GammaGap ≥ 3.65 (g/dL)			
Models ^a^	Tertiles of MAMC	Hazard Ratio (95% CI)	*P* -value	Models ^a^	Tertiles of MAMC	Hazard Ratio (95% CI)	*P* -value
**Male**				**Male**			
Model 1	T2 v.s. T1 T3 v.s. T1	0.46 (0.34–0.61) 0.20 (0.15–0.27)	< 0.001 < 0.001	Model 1	T2 v.s. T1 T3 v.s. T1	0.40 (0.24–0.67) 0.17 (0.10–0.28)	< 0.001 < 0.001
Model 2	T2 v.s. T1 T3 v.s. T1	0.95 (0.71–1.28) 0.76 (0.56–1.04)	0.759 0.082	Model 2	T2 v.s. T1 T3 v.s. T1	0.67 (0.39–1.14) 0.51 (0.29–0.90)	0.141 0.021
Model 3	T2 v.s. T1 T3 v.s. T1	0.93 (0.69–1.25) 0.73 (0.53–1.00)	0.639 0.049	Model 3	T2 v.s. T1 T3 v.s. T1	0.55 (0.31–0.96) 0.40 (0.22–0.73)	0.035 0.003
Model 4	T2 v.s. T1 T3 v.s. T1	0.98 (0.72–1.32) 0.75 (0.54–1.02)	0.870 0.070	Model 4	T2 v.s. T1 T3 v.s. T1	0.48 (0.27–0.85) 0.37 (0.20–0.69)	0.012 0.001
**Female**				**Female**			
Model 1	T2 v.s. T1 T3 v.s. T1	1.45 (1.20–1.75) 1.68 (1.22–2.31)	< 0.001 0.001	Model 1	T2 v.s. T1 T3 v.s. T1	0.97 (0.69–1.36) 1.55 (1.01–2.38)	0.862 0.043
Model 2	T2 v.s. T1 T3 v.s. T1	1.06 (0.88–1.28) 1.79 (1.30–2.47)	0.529 < 0.001	Model 2	T2 v.s. T1 T3 v.s. T1	0.70 (0.50–0.98) 1.16 (0.75–1.78)	0.040 0.502
Model 3	T2 v.s. T1 T3 v.s. T1	1.06 (0.88–1.28) 1.80 (1.30–2.49)	0.521 < 0.001	Model 3	T2 v.s. T1 T3 v.s. T1	0.68 (0.49–0.96) 1.08 (0.70–1.67)	0.027 0.718
Model 4	T2 v.s. T1 T3 v.s. T1	1.06 (0.88–1.28) 1.77 (1.28–2.45)	0.559 0.001	Model 4	T2 v.s. T1 T3 v.s. T1	0.68 (0.48–0.96) 1.09 (0.71–1.69)	0.028 0.695

^a^Adjusted covariates:

Model 1 = Unadjusted.

Model 2 = Model 1 + age, race and body mass index (BMI).

Model 3 = Model 2 + serum high-density lipoprotein (HDL), serum fasting glucose, serum total cholesterol , serum total bilirubin, serum aspartate aminotransferase (AST).

Model 4 = Model 3 + history of congestive heart failure (CHF), stroke, malignancy and smoking.

Increasing MAMC tertiles were significantly associated with decreasing HRs for all-cause mortality among 60–90 years old individuals with gamma gap ≥ 3.65 g/dl (Table 2) and those with BMI 19-24.9 kg/m^2^ and gamma gap ≥ 3.65 g/dl (Table 3). Moreover, in male participants, the MAMC was in significant association with decreasing HRs for all-cause mortality regardless of the gamma gap levels (Table 4).

No statistically significant correlation was noted between MAMC and cancer mortality in both gamma gap groups stratified by age (Table 5). Increasing MAMC tertiles were statistically significant associated with lower HRs for cancer mortality in male participants with gamma gap≥ 3.65 g/dl (Table 6) and in participants with BMI 19-24.9 kg/m^2^ and gamma gap ≥ 3.65 g/dl (Table 7).

Participants aged 60–90 years with gamma gap ≥ 3.65 g/dl (Table 8), those with BMI 19-24.9 kg/m^2^ and gamma gap ≥ 3.65 g/dl (Table 9), and male participants in group with gamma gap ≥ 3.65 g/dl (Table 10) had significantly positive association between higher MAMC tertiles and lower HRs for CV mortality. Furthermore, individuals aged 20–39 years with gamma gap < 3.65 g/dl also had the identical association between MAMC and CV mortality.

According to Tables 2–10, in the group with gamma gap < 3.65 g/dl, the association between the MAMC tertiles and HRs for all-cause mortality, cancer mortality, and CV mortality were lack of statistical significance.

In conclusion, while the MAMC tertiles increased in the elevated gamma gap group (gamma gap ≥ 3.65 g/dl), individuals with elder age (60–90 years), normal range of BMI (19-24.9 kg/m^2^), and male gender tended to have lower HRs for all-cause mortality, cancer mortality, and CV mortality. The results indicated higher MAMC may be a protective factor of all-cause mortality, cancer mortality, and CV mortality.

## DISCUSSION

We discovered a statistical significant lowering down of HRs of mortality in higher MAMC tertiles among older male participants with normal BMI values and within the group with gamma gap ≥ 3.65 g/dl. This results showed that higher MAMC may possess protective power over all-cause mortality, cancer mortality, and CV mortality in normal weight older male people with gamma gap ≥ 3.65 g/dl.

Previous literature suggested that MAMC could assist biochemical analysis to characterize undernutrition status [3]. MAMC could represent muscle mass due to its strong correlation with the accurate dual-energy X-ray absorptiometry (DEXA) [4]. Higher MAMC was an indicator of larger lean body mass in maintenance hemodialysis (MHD) patients and was also a potential predictor of better quality of life and survival rate [4]. Combined use of both waist circumference (WC) and MAMC provided simple measures of body composition to assess mortality risk in older men [5]. Moreover, Landi *et al.* reasoned that decreased MAMC was associated with mortality in elderly men and women [6].

Gamma gap is also known as paraprotein gap or globulin gap [7]. High gamma gap has important implications of inflammatory states [8, 9, 10]. Albumin, the most abundant protein in plasma, serves as a circulating depot for endogenous and exogenous compounds [11]; however, viral infection, malignancies and autoimmune diseases lead to excessive production of immunoglobulins, which raise the level of gamma gap independent of albumin [12]. Despite only few surveys applies gamma gap to predict clinical outcomes, the operational definition of an elevated gamma gap is above 4.0 g/dl [1]. Among hospitalized patients, hypoalbuminemia is a common sign in reflection of inflammation [13]. In addition, other cytokines in inflammatory process stimulate acute-phase proteins production and result in elevation of gamma gaps. Hyperglobulinemia accompanied by hypoalbuminemia is a frequent condition in chronic autoimmune disease [14]. Patients suffering from multiple myeloma or other immunoproliferative disease has been known to show gross divergences of serum proteins constitution in comparison with normal people, including hyperproteinemia and associated change in albumin-globulin ratio (AGR) [15]. A large retrospective cohort study conducted in Korea indicates that subjects with low AGR are at risk for increased all-cause mortality, cancer mortality, and cancer incidence [16]. Juraschek *et al.* asserts gamma gap is in strong association with all-cause mortality and specifically, death from pulmonary diseases [1]. After taking physical activity into consideration, Loprinzi *et al.* illustrate that moderate-to-vigorous physical activity (MVPA) reduces death from any cause among people with elevated gamma gaps [17].

Due to the correlation between high gamma gaps and inflammation, Juraschek et al. speculate that elevated gamma gaps have strong association with mortality [1].

Physical activity has salient influences in lowering down all-cause mortality [18]. Routine exercise modulates body composition through controlling weight and attenuating abdominal adiposity [19, 20]. It can enhance lipid profiles [21, 22], insulin sensitivity [23, 24], cardiac function [25, 26] and improve autonomic tone [27] and inflammatory states [28]. During an inflammation process, activated cells produce cytokines, including interleukin-6 (IL-6), interleukin-1β(IL-1β), tumor necrosis factor a (TNF-α), interferon-γ (IFN-γ), and transforming growth factor-β (TGF-β). These pro-inflammatory cytokines impede normal physical function by production of oxygen free radicals, apoptosis, and activation of leukocytes [29, 30, 31]. Appropriate exercise affects beneficially the inflammatory cytokines, and cause reduction of IL-6 [32], TNF-α [33], and IFN-γ [34]. Considering the above mentioned, we assume appropriate exercise possesses moderation effects on inflammation and plays a major role in lowering down mortality risk among population with high gamma gap. This conclusion is in consistent with Loprinzi *et al.*, who suggests physical activity is beneficial in mortality among patients with high levels of gamma gap [17].

However, evaluating exercise requires standardized questionnaires, even trained personnel, and current investigations utilize different methods to acquire data. These high heterogeneity surveys can engender bias in future performing meta-regression analyses. Therefore, we use measurable anthropometric data in this study to reflect physical activity. As physical activity is associated with muscle mass [35], and MAMC can well represent muscle mass [4], we employed the MAMC to predict mortality of people with normal and elevated gamma gap in this survey. Noteworthy, in the group with gamma gap ≥ 3.65 g/dl, the HRs of all-cause mortality, cancer mortality, and CV mortality decreased as MAMC increased among older male participants. Genetic factors, hormones effects, muscle capacity and physical function may explain for the presence of gender differences of the results [36, 37]. Generally, men have higher muscle mass and muscle capacity than women due to hormone effects, such as much higher levels of testosterone in men. Males also have greater muscle strength and higher physical performance than females [36, 37].

The study does have a number of caveats. First, NHANES III is a study with single measurement of MAMC during the follow-up period, which may engender biased results. The present study is only having exposure data available at one time point, future work should investigate how altered elevated gamma gap and MAMC influences mortality. Second, a myriad of the clinical factors used in the investigation are based on household claimed data that may not be completely accurate. Third, despite adjustments having been made for a large number of potentially confounding factors, unmeasured confounders of the association between MAMC and cause specific mortalities in US individuals cannot be ruled out.

Despite the aforementioned limitations, the present study corroborates previous work demonstrating an positive correlation between all-cause mortality, cancer mortality, CV mortality and those with gamma gap ≥ 3.65 g/dl, also providing evidence supporting the protective effect of higher MAMC on mortality among individuals with an elevated gamma gap. Future work may benefit by examining the extent to which changes in MAMC influence mortality risk among people with an elevated gamma gap. Future work would also benefit by applying our findings to clinical and epidemiological investigations.

## MATERIALS AND METHODS

### Data source and participants

Data were retrieved from the NHANES III (1988–1994), a cross-sectional survey designed to evaluate the health and nutritional status of the noninstitutionalized U.S. adults by the National Center for Health Statistics (NCHS) of the Centers for Disease Control and Prevention (CDC) [38]. Approved by Institutional Review Board (IRB), NHANES III comprised household interviews and physical examinations conducted at mobile examination centers. The NHANES III survey was based on a complex, multistage, stratified and clustered design and was executed in accordance with the Declaration of Helsinki. We selected adults between 20 to 90 years of age in the NHANES III to performed the present study, and these participants were involved with a mean follow-up of 14.3 years.

### Follow-up data on all-cause mortality, cancer mortality, and cardiovascular mortality

NCHS linked NHANES III survey to death certificate records with the National Death Index (NDI), a computerized database of all certified deaths in the U.S. since 1979 [39]. Linkage of the NHANES III participants with the NDI mortality data provided the opportunity to conduct a vast array of outcome surveys designed to investigate the correlation of health factors with mortality. This file linked NHANES III participants with death records from the NDI through 31 December 2006. The cause of death was coded using the International Classification of Disease (ICD)-9 until 1998 and ICD-10 was used from 1999 onward. To adjust for changes between the two coding systems, final cause of deaths occurring prior to 1999 were re-coded into comparable ICD-10-based underlying cause of death groups [40]. For overall mortality, we included deaths from all causes; for cancer-specific mortality, we included deaths from malignant neoplasms (ICD-10 = C00–C97); for CV diseases related mortality, we included diseases of the heart and circulation system (ICD-10 = I00–I178).

### Measurement: gamma gap

According to the original protocol, NHANES III collected a comprehensive serum metabolic data form the participants. The analyses were using the Hitachi Model 704 multichannel analyzer. The NHANES personnel evaluated total protein with a colorimetric assay, while albumin was determined via a Bromocresol purple reagent. Gamma gap was calculated with the following formula: serum total protein (g/dl)–serum albumin (g/dl). In clinical practice, the threshold of gamma gap was 3.5 g/dl or 4.0 g/dl [1]. We conducted the ROC curve for gamma gap. The area under the curve (AUC) was an index of the ability of a marker to discriminate between true positives and true negatives. In the present study, an elevated gamma gap was defined using the cut-point of ≥ 3.65 g/dl.

### Measurement: anthropometric parameters

The anthropometric parameters were undertaken using a standard protocol and collection instruments as outlined below. Body height and weight were measured, and converted to BMI in units of kg/m^2^. Have the participants standing upright with relaxed shoulder, and marked at the midpoint posteriorly to the upper arm. Then placed a tape measure around the target point and pressed to the skin surface without tight compress. The operator measured the circumference of the upper arm vertically to the long axis of it. The MAC value was measured to the nearest 0.1 cm. To measure the TS, the operator grasped about 2.0 cm of the TS above the marked point and kept the jaws of the skinfold calipers vertically to the shaft of the arm over the marked point. The TS value was recorded to the nearest 0.1 mm. MAMC (cm), an established measure of muscle protein mass, was calculated as: MAC (cm) − 0.3142 x TS thickness (mm).

### Definition of the MAMC tertiles group

We categorized both male and female participants into tertiles based on their MAMC level. The participants in the lowest tertile was defined as the reference group. The gender-specific tertiles were as follows: T1 (15.9–27.3), T2 (27.4–29.7), T3 (29.8–40.9) cm in the male group and T1 (13.8–21.9), T2 (22.0–24.2), T3 (24.3–44.1) cm in the female group.

### Measurement: risk variables

Self-report variables were as the below mentioned: age, gender, ethnicity, smoking status, past medical history diagnosed by a doctor (type 2 DM, skin cancer, other cancer, stroke, CHF, and asthma).

Metabolic variables were obtained from blood samples. The hexokinase enzymatic method was adopted to analyze the plasma glucose according to the Cobas Mira Chemistry System (Roche Diagnostic Systems, Indianapolis, IN, USA). The venipuncture time of the participants was after fasted for 6 hours. Serum TC, serum TG, serum HDL and serum LDL were measured by the Hitachi 704 Analyzer (Roche Diagnostics, Indianapolis, IN, USA). Serum CRP level was measured with latex-enhanced nephelometry (Behring Nephelometer II Analyzer System; Behring Diagnostics Inc., Somerville, NJ, USA). The study utilized the Beckman Synchron LX20 instrument to measure other biochemical profiles, such as serum albumin, serum UA, serum total bilirubin, AST, and ALT. Moreover, MAMC, gamma gap, BMI, SBP, and DBP were listed as continuous variables. The database had past the appraisal of the CDC, and all the profiles were obtained under standardized protocols.

### Statistical analysis

Predictive Analytics Suite Workstation Statistics (SPSS Inc., Chicago, IL, USA) (name as SPSS hereinafter) is an integrated software program that addresses the entire analytical process, from planning to data collection to analysis, reporting and deployment. NHANES III was a database with complex survey designs; thus, it was inappropriate to calculate statistical analyses with the assumption of a simple random sample providing incorrect variance estimates. ‘‘Complex Sampling’’ was used to incorporate sample weights and adjusted for strata of the complex sample design. Continuous data are presented as means ± standard errors (SE) while categorical data as count and percentages (%). To examine the effect of MAMC and gamma gap on mortality outcomes, we would describe the difference in mortality outcomes between MAMC as a main effect. Similarly, any difference in the level of gamma gap would be presented as a main effect. The presence of an interaction effect implies that the effect of MAMC on mortality outcomes varies as a function of the level of gamma gap. As a first step, the analysis of pooled MAMC values versus pooled gamma gap levels was performed. If positive, interaction tests were performed on all-cause mortality, cancer mortality, and CV mortality to check for any interaction between the different predicting components (MAMC, gamma gap). Independent of the interaction test, an assessment of predicting effect was also performed. An interaction could be ruled out if the statistical interaction test was not significant and the assessment revealed no relevant difference. If an interaction was ruled out, the pooled analysis remained the primary analysis. If an interaction could not be ruled out, then we would divide the gamma gap into subgroups. For further analyses, the ROC curves of gamma gap for detecting all-cause mortality, cancer mortality, and CV mortality were produced. The AUCs with their 95% CIs were calculated. To determine the optimal point, the square root of [(1 – sensitivity)^2^ + (1 – specificity)^2^] was calculated, which was the point on the ROC curve with the shortest distance from the upper left corner. The study would divide the participants into subgroups according to the cut-off values of gamma gap and measure the influences of MAMC stratified by age (20–39, 40–59, and 60–90 years), BMI (19–24.9, 25–29.9, and ≥ 30 kg/m^2^), and gender on all-cause mortality, cancer mortality and CV mortality.

Table 1 illustrated the demographic characteristics, and the continuous variables were analyzed by trend analyses, while the categorical variables were analyzed by Chi-square test. *P* values of less than 0.05 were considered significant.

Multivariable Cox proportional hazard ratio models stratified by age, BMI, and gender were used to explored the HRs between the MAMC tertiles ratios (T2 v.s. T1 and T3 v.s. T1) and all-cause mortality (Tables 2–4), cancer mortality (Tables 5–7), and CV mortality (Tables 8–10) in the two gamma gap groups. The multivariable Cox proportional hazard ratio models were classified into 4 models that adjusted for the following variables:

Model 1: unadjusted by other variables.
Model 2: age, gender, race and BMI.
Model 3: age, gender, race, BMI, serum HDL, serum fasting glucose, serum TC, serum total bilirubin and AST.
Model 4: age, gender, race, BMI, serum HDL, serum fasting glucose, serum TC, serum total bilirubin, AST, CHF, stroke, malignancy and smoking.
